# Multiple adult xanthogranuloma^☆^^☆☆^

**DOI:** 10.1016/j.abd.2019.02.010

**Published:** 2019-12-18

**Authors:** Renata da Costa Almeida, Óscar Tellechea, Mariana Pinho Pereira, Rosa Cristina Correia Mascarenhas

**Affiliations:** a Physician Assistant in Family Medicine, Portugal; b Dermatology Service, Centro Hospitalar Universitário de Coimbra, Coimbra, Portugal; c Dermatology Service, Hospital Distrital da Figueira da Foz, Figueira da Foz, Portugal; d Physician Assistant in Family Medicine, Unidade de Cuidados de Saúde Primários Litoral, Alfeizerão, Portugal

Dear Editor,

Xanthogranuloma (XG) is a normolipemic non-Langerhans cell histiocytosis (NLCH) most commonly seen in childhood and generally designed as juvenile xanthogranuloma (JXG). Infrequently, XG can occur in adulthood. Both in adults and children, XG usually presents as a solitary lesion. Multiple lesions are rare in JXG and exceptional in adults. The authors report a case of multiple adult xanthogranuloma (MAXG) of the face, neck, trunk, abdomen, and axillae, with no extracutaneous involvement and no association with hematologic disease.

A 38-year-old female, with no relevant personal or family history of the disease, was seen in October 2016 with multiple papules on her skin, which she first noticed 18 months before. Dermatologic examination showed more than 100 yellowish-brown, smooth, firm papules with diameters of 1–3 mm on the face, neck, thorax, abdomen, and axillae (Fig. 1), two larger elements, standing out in the outer corner of the right eye and in the homolateral nasogenian groove. The lesions were asymptomatic and the general physical examination (including ophthalmologic, cardiopulmonary, and neurologic) was normal.

**Figure 1:**
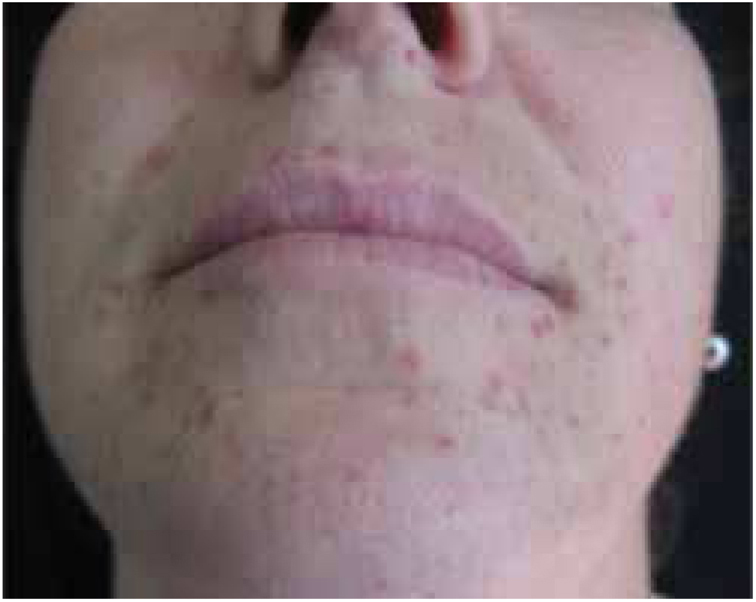
Multiple xanthogranulomas. Detail of yellowish-brown, smooth, firm papules with diameters of 1–3 mm, asymptomatic, located on the face.

The diagnostic hypotheses of syringomas, sarcoidosis, xanthomas, and histiocytosis were considered, and two lesions were excised for histopathologic evaluation. This disclosed, in both specimens, a circumscribed dense dermal cellular infiltrate composed of large histiocytes, mostly exhibiting xanthomized cytoplasm, accompanied by multinucleated giant cells, including some of the Touton type. Interspersed inflammatory cells were also present, mostly small lymphocytes with occasional neutrophils and some eosinophils (Figure 2, Figure 3). Immunohistochemical evaluation documented absence of S100 protein, and CD1a immunoreactivity and diffuse CD68 positivity, supporting the diagnosis of XG.

**Figure 2:**
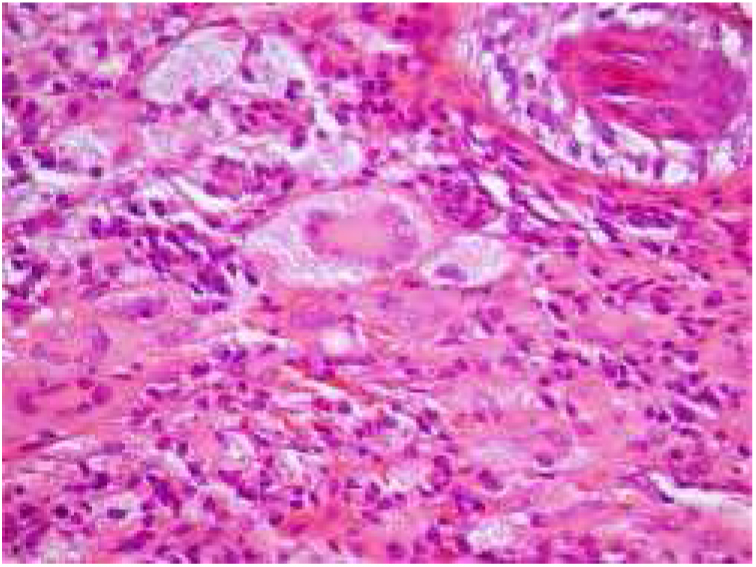
Infiltration circumscribed of the dermis by xanthomized histiocytes and multinucleated Touton giant cells (Hematoxylin & eosin, ×400).

**Figure 3:**
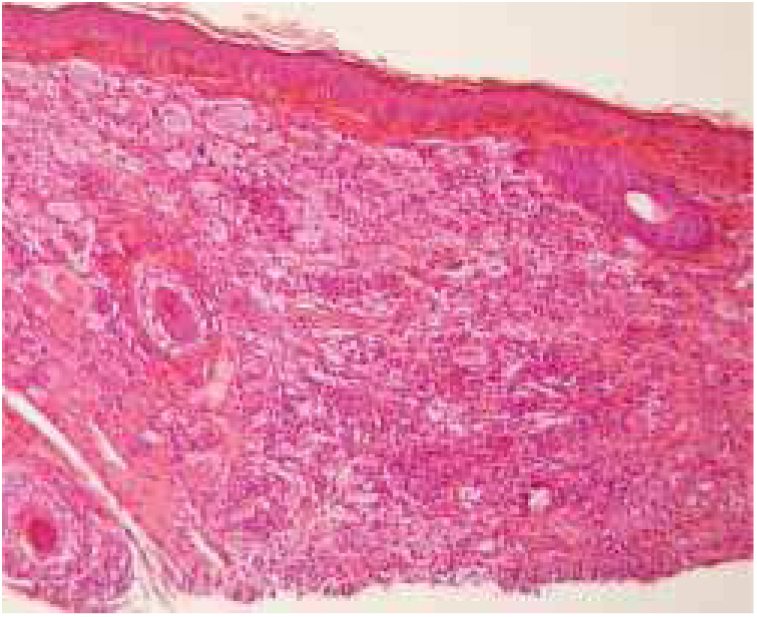
Diffuse histiocytic proliferation in the superficial and deep dermis associated with lymphocytic infiltrate and with multinucleated Touton giant cells (Hematoxylin & eosin, ×100).

General laboratorial and imaging evaluation (including β2 microglobulin, serum protein electrophoresis, skeletal and thorax X-ray, and abdominopelvic echography) showed no abnormalities. On the basis of the clinico-laboratorial and imaging findings, the diagnosis of MAXG was obtained. Ablation of the larger and cosmetically more-disturbing lesions by CO_2_ laser or electrosurgery was performed, with good results. During the follow-up period of almost two years, the untreated cutaneous lesions persisted, with no development of extracutaneous lesions nor hematologic alterations.

XG is the most common type of NLCH.^1^ The presentation of XG in adulthood is infrequent, occurring generally in the 3rd or 4th decades of life, without gender predominance.^2^ As in JXG, most cases of adult XG manifest as a solitary papule or nodule on the face.^3^ The occurrence of multiple XG lesions in adults is rare. MAXG is defined by more than five XGs in patients over 14 years of age.^2^ According to these criteria, 118 cases of MAXG have been reported from 1969 to 2004.^3^ The etiopathogenesis of MAXG is unknown.^3^

JXG and MAXG exhibit identical histologic and immunohistochemical findings.^1^ Mature lesions show, as in the present case, a circumscribed nodular dermal proliferation composed of xanthomized histiocytes, characteristically accompanied by Touton multinucleated cells.^1^, ^4^ The histiocytic nature of the proliferation is confirmed by their CD68 positivity and absence of S100 protein, as well as CD1a immunoreactivity, with variable expression of factor XIIIa.^4^, ^5^ Nevertheless, these microscopic and immunochemical findings are not pathognomonic for XG and, in the present patient, other forms of adult NLCH presenting with multiple lesions needed to be excluded, namely Erdheim–Chester disease (ECD), as well as eruptive disseminated histiocytoma (EDG), and more remotely, disseminated xanthoma (DX) and multicentric reticulohistiocytosis (RM).^4^, ^5^

The absence of skeletal abnormalities allowed to exclude ECD. In EDG, the lesions do not contain Touton cells. These can, however, occur in DX, but in the present patient no linear periflexural lesions, which characterize DX, were present. In RM cutaneous lesions are acrally located, accompanied by arthropathy and microscopically characterized by large multinucleated ground-glass cells, aspects absent in the present case.^4^, ^5^

Clinically, MAXG presents as yellow-orange or brownish papules and/or nodules distributed on the trunk, face, neck, and less frequently, the limbs. More than 10 lesions are present in only about 6% of the reported cases.

In contrast with JXG, in which ocular and, less frequently, other organ involvement may occur, MAXG does not seem to evolve with extracutaneous histiocytic infiltration. Accordingly, exhaustive systemic investigation is not recommended in every patient. Nevertheless, as in JXG, MAXG can be a marker of malignant blood disease, a possibility that should be considered whenever B symptoms and/or alterations of the electrophoretic proteinogram occur.^3^, ^4^, ^5^ These, as well as full blood tests, were systematically absent/negative in the present patient during the almost two years of follow-up.

As spontaneous regression of the lesions in MAXG is less probable than in JXG, surgical excision, electrosurgery, ablation by CO_2_ laser, or systemic retinoids – namely isotretinoin – have been recommended as treatments whenever lesions are numerous, cause discomfort, or are unesthetic. In the present patient, a female of child-bearing age, the larger papules were treated iteratively with CO_2_ laser or electrosurgery, with a good cosmetic result.

In conclusion, MAXG is a rare type of adult normolipemic NLCH, whose significance is insufficiently known, and is occasionally associated with hematologic malignancy that occur simultaneously, preceded by or following the diagnosis of this histiocytosis,^3^ thereby requiring a focused and long-term follow-up of the patients.

## Financial support

None declared.

## Authors’ contribution

Renata da Costa Almeida: Approval of the final version of the manuscript; composition of the manuscript; design of the study; critical review of the literature; critical review of the manuscript.

Óscar Tellechea: Approval of the final version of the manuscript; composition of the manuscript; design of the study; critical review of the literature; critical review of the manuscript.

Mariana Pinho Pereira: Approval of the final version of the manuscript; composition of the manuscript; design of the study; critical review of the literature; critical review of the manuscript.

Rosa Cristina Correia Mascarenhas: Approval of the final version of the manuscript; composition of the manuscript; collection, analysis, and interpretation of data; design of the study; critical review of the literature; critical review of the manuscript.

## Conflicts of interest

None declared.
